# Forty years after Alma-Ata: primary health-care preparedness for chronic diseases in Mozambique, Nepal and Peru

**DOI:** 10.1080/16549716.2021.1975920

**Published:** 2021-09-27

**Authors:** Maria Kathia Cárdenas, Silvana Pérez-León, Suman Bahadur Singh, Tavares Madede, Savaiva Munguambe, Valério Govo, Nilambar Jha, Albertino Damasceno, J. Jaime Miranda, David Beran

**Affiliations:** a CRONICAS Centre of Excellence in Chronic Diseases, Universidad Peruana Cayetano Heredia, Lima, Peru; b B. P. Koirala Institute of Health Sciences, Dharan, Nepal; c Faculty of Medicine, Eduardo Mondlane University, Maputo, Mozambique; d Research Unit, Department of Internal Medicine, Maputo Central Hospital, Maputo, Mozambique; e School of Medicine, Universidad Peruana Cayetano Heredia, Lima, Peru; f Division of Tropical and Humanitarian Medicine, Faculty of Medicine, University of Geneva, Geneva University Hospitals, Geneva, Switzerland

**Keywords:** Health systems, primary health-care, chronic conditions, developing countries

## Abstract

**Background:**

Four decades after the Alma-Ata Declaration, strengthening primary health care (PHC) remains a priority for health systems, especially in low- and middle-income countries (LMICs). Given the prominence of chronic diseases as a global health issue, PHC must include a wide range of components in order to provide adequate care.

**Objective:**

To assess PHC preparedness to provide chronic care in Mozambique, Nepal and Peru, we used, as ‘tracer conditions’, diabetes, hypertension and a country-specific neglected tropical disease with chronic sequelae in each country.

**Methods:**

By implementing a health system assessment, we collected quantitative and qualitative data from primary and secondary sources, including interviews of key informants at three health-system levels (macro, meso and micro). The World Health Organization’s health-system building blocks provided the basis for content analysis.

**Results:**

In total, we conducted 227 interviews. Our findings show that the ambitious policies targeting specific diseases lack the support of technical, administrative and financial resources. Data collection systems do not allow the monitoring of individual patients or provide the health system with the information it requires. Patients receive limited disease-specific information. Clinical guidelines and training are either non-existent or not adapted to local contexts. Availability of medicines and diagnostic tests at the PHC level is an issue. Although medicines available through the public health care system are affordable, some essential medicines suffer shortages or are not available to PHC providers. This need, along with a lack of clear referral procedures and available transportation, generates financial issues for individuals and affects access to health care.

**Conclusion:**

PHC in these LMICs is not well prepared to provide adequate care for chronic diseases. Improving PHC to attain universal health coverage requires strengthening the identified weaknesses across health-system building blocks.

## Background

In 1978, the Alma-Ata Declaration [1] called for strengthening primary health care (PHC) as an integral part of developing the health system and the community’s role in ensuring healthy populations. Four decades later, most of the evidence about the role of the PHC comes from high-income countries [2]; nonetheless, strengthening PHC remains a priority for health systems in low- and middle-income countries (LMICs) [3]. Given the epidemiological transition, with the increase in noncommunicable diseases (NCDs) [4,5] in parallel with the burden of communicable diseases, PHC must be able to deliver care for all health-related needs of the population it serves [6]. One specific concern in health systems in LMICs is the lack of capacity to manage chronic long-term conditions that NCDs or communicable diseases cause [7–10]. PHC is key to addressing the supply of quality health services and the demand factors influencing services the population utilises [11]. Although the Sustainable Development Goals (SDGs) do not directly mention PHC [12], the 2018 Declaration of Astana highlighted its relevance: ‘PHC is a cornerstone of a sustainable health system for universal health coverage and health-related SDGs’ [13].

The causes of NCDs and neglected tropical diseases (NTDs) differ, but both require chronic disease management with a focus on continuity of care, regular access to medicines and health services and appropriate patient education [14–16]. This study aims to assess PHC preparedness to provide chronic care in Mozambique, Nepal and Peru,using specific NCDs (diabetes and hypertension) and respective country-specific NTDs as ‘tracer conditions’ [17,18]. The rationale for using two distinct groups of diseases rests on their potential for identifying ‘strengths and weakness of services delivery and health systems’ [19]. Thus, the study of ‘tracer conditions’ as a tool for evaluating PHC preparedness creates a unique framework for explaining the challenges of chronic care in LMIC settings.

## Methods

This study utilised a Rapid Assessment Protocol [20], implemented in various LMICs and adapted for this study [21–23]. The methodology took a mixed-methods approach, included various types of informants at different levels of the health system (macro, meso and micro) and relied on multiple data sources and topics of study. We gathered secondary data and collected primary data from interviews. The selected ‘tracer conditions’ were diabetes and hypertension (NCDs) for all countries and a country-specific NTD: schistosomiasis (Mozambique), leprosy (Nepal), and neurocysticercosis (Peru).

## Settings

We implemented the study in rural and semi-urban communities in Mozambique, Nepal and Peru [24]. These LMICs represent three different geographical, socio-economic and health-system contexts (Table 1).

**Table 1.** General information of Mozambique, Nepal and Peru

Indicator	Mozambique	Nepal	Peru
Country income group ^(1)^	Low income	Low income	Upper middle income
Population (in thousands, year 2018) ^(1)^	29,496	28,088	31,989
Gross Domestic Product (GDP) per capita in US current dollars (year 2018) ^(1)^	503.3	1,038.7	6,941.2
Life expectancy at birth (year 2018) ^(1)^	60.2	70.5	76.5
Current health expenditure (% of GDP, year 2018) ^(2)^	8.2	5.8	5.2
Physicians (per 10,000 people, year 2018) ^(2)^	0.8	7.5	8.2
Age-standardized NCD mortality rate (per 100,000 population, year 2018) ^(2)^	783.6	602.3	305.5
Districts with PHC facilities included in the study (population)	Moamba (83,879) [25] Nlhamankulu (129,306) [25]	Baniyani (6,458) [26] Itahari (140,517) [27]	Montero (6,179) [28] Ayabaca (30,852) [28]

(1) World Development Indicators [29]; (2) Global Health Observatory data repository [30].

## Sampling

We purposively selected and enrolled 10 PHC facilities (4 in Mozambique, 4 in Peru and 2 in Nepal) based on the following criteria: 1) At least half of PHC facilities are located in rural low-income areas; 2) Hypertension and diabetes are present in the selected PHC facility areas, and the selected NTD is endemic in the respective study setting; 3) Adequate accessibility (e.g. feasible to reach using public transportation) to each PHC facility; 4) PHC facilities do not participate in other research projects; 5) The head of each PHC facility is willing to participate in the study.

The selection of participants was purposively and followed the ‘snowball’ technique until reaching data saturation. Interviewees were key stakeholders at the national (macro) and intermediate (meso) level due to their role in the health system with experience in NCDs or NTDs; or they were patients (or caregivers) living with one or more selected diseases or health care workers managing people with NCDs or NTDs (micro-level). Macro-level interviews included individuals from the national Ministry of Health, disease-specific associations or organizations, and clinical or academic opinion leaders. Targeted participants at the meso-level were from regional and local health offices, public and private laboratories, public and private pharmacies and heads of PHC facilities. Finally, micro-level interviews focused on PHC workers (physicians, nurses, obstetricians, etc.) and people (or caregivers) living with NCDs or NTDs.

## Data collection

A questionnaire in English was locally adapted by each country team and translated to Spanish in Peru, Portuguese in Mozambique and English in Nepal. Each country team conducted interviews in the following languages: Spanish (Peru); Portuguese and Changane (Mozambique); Nepali, Hindi and Maithali (Nepal). The fieldwork team in each country, composed of five to seven members, had a background in health sciences, previous experience conducting interviews in community-based projects and the ability to communicate in the local languages.

One or two researchers in each country trained the fieldworkers in their local official language. The training took over 20 hours and also included the piloting of the interview tools. Other topics covered during the training included NCDs and NTDs, interviewing techniques, application of tools, ethics, research topics and data entry. The fieldwork team conducted all micro-level interviews. At the macro and meso levels, one or two researchers conducted the interviews. All interviews took place between December 2016 and August 2017.

The interviews addressed different topics of study: 1) National health plans, policies, strategies, or action plans for preventing and managing NCDs and NTDs; 2) Organizational structure within the Ministry of Health related to the selected NCDs and NTDs; 3) National budget line for the selected NCDs and NTDs; 4) Funding of the selected diseases and financial consequences for patients; 5) National surveillance system and registry of the selected diseases; 6) National clinical practice guidelines about prevention and management of the selected diseases at the PHC level; 7) Knowledge and training of health-care workers on the selected diseases; 8) Availability and price of tests and consumables for the selected diseases; 9) Availability and price of essential medicines for the selected diseases; 10) Prevention of the selected diseases through patient education; 11) Referral process information and implementation. In addition to these topics, interviews also included other questions to inform about potential interventions in each country [24,31].

Each interviewer recorded the information manually and entered it using a form in Microsoft Excel. Only a small proportion of interviews were recorded, especially those related to the macro-level and some health-care workers. The information was quantitative (e.g. quantity and price of medicines, equipment, and consumables) and qualitative (knowledge and opinion on specific topics). We confirmed the availability of medicines through on-site observation on the day of the visit. We recorded the retail prices of medicines at the public PHC pharmacy and private pharmacies located near the PHC. Secondary data sources, mainly policy documents and health reports, were provided by the participants or gathered from public sources.

A list of the country-specific policy documents and secondary sources collected is presented in Supplementary material 1. We also prepared a data collection and analysis matrix (Supplementary material 2) to have a standard structure of topics covered and questions used in all countries, thus facilitating cross-country comparison. We also used the World Health Organization (WHO)’s recommendations on monitoring and evaluation framework [32] and a manual to investigate NCDs in LMICs [20] to identify the topics and core information to include in the matrix.

## Data analysis

Using the findings from interviews (quantitative and qualitative) and secondary sources (policy documents, reports, technical documents), we conducted a content analysis applying the WHO’s health-system building blocks [33]. The matrix that shows the data collection and analysis (Supplementary material 2) presents the collected information, identifies the relevant WHO building block and notes the sources and questions. We used the matrix to follow a schematic process, to organise data collection and facilitate data analysis and cross-country comparisons of the selected diseases at the PHC level. We triangulated and combined findings from different types of informants, various types of information and multiple sources, to corroborate the information and show the informants’ perspectives. Researchers from a different country team reviewed the results of a country other than their subject country, to contribute to the reflexive process.

The qualitative data came from open-ended questions designed to provide a deeper understanding of the topics of study. After reading and re-reading the qualitative data using the Microsoft Excel database, each country team prepared a summary of each topic. Some interviews were recorded, enabling the extraction of verbatim quotes. Quantitative data analysis used proportions for the availability of medicines and median prices to measure affordability. We applied the methodology proposed by Health Action International and WHO to assess affordability [34], calculating the monthly cost of medicines and the number of days at the lowest-paid government worker’s daily wage that would cover it. First, assuming 30 days per month, we calculated the monthly treatment cost for each medicine, using its median price for the ‘Defined Daily Dose’ in the surveyed facilities [35]. Then, we divided the monthly treatment cost by the monthly wage for the lowest-paid government worker in each country, using the following monthly wages for 2018: 4,255 Mozambican Metical in Mozambique, 16,230 Nepalese Rupee in Nepal and 930 Peruvian Sol in Peru.

## Ethics

Participation in the study was voluntary, informed consent was sought, and ethical approval was obtained from: Commission Cantonale d’éthique de la recherche (Switzerland); Faculdade de Medicina/Hospital Central de Maputo (Mozambique); Nepal Health Research Council (Nepal); and Universidad Peruana Cayetano Heredia (Peru). After receiving the ethical approval, each country team also requested authorization from the concerned health authorities and PHC facilities.

## Results

In total, we conducted 227 interviews, averaging 60 minutes each. (Table 2) The WHO building blocks structure the presentation of the interview results and secondary data [33].

**Table 2.** Number of interviews conducted per type of informant at the macro, meso and micro-level

Health system level	Mozambique	Nepal	Peru
***Macro-level interviews****: Ministry of Health, disease-specific national associations or organizations and key opinion leaders*	9	9	25
***Meso-level interviews****: Regional and local health offices, heads of PHC facilities, public/private laboratories and pharmacies*	20	16	19
***Micro-level interviews****: Health-care workers (physicians, nurses, obstetricians, etc.) and people with NCDs or NTDs*	28	46	54
**Total number of interviews:**	**57**	**72**	**98**
**Number of PHC facilities:**	**4**	**2**	**4**

### Governance

Each country had established a functioning unit or department in the Ministry of Health responsible for NCDs: Mozambique in 2000, Nepal in 2009 and Peru in 2004. All NCD departments had shortages of human and financial resources. According to some policy makers in Mozambique, NCDs had received much less attention and insufficient resources than programmes for malaria or HIV. These inadequacies also characterised the NTD departments in Mozambique and Peru. In Mozambique, the NTD department covered schistosomiasis; in Peru, the remit of the NTD department did not specifically include neurocysticercosis. Nepal’s Leprosy Control Division, several decades old, had received substantial resources from international partners.

Despite the inclusion of NCDs in various strategic documents, some macro-level participants in the three countries mentioned insufficient resources and low priority of the PHC on addressing NCDs as primary pitfalls. Overall, considering the available resources, plans were called ‘ambitious’. Mozambique and Peru had implemented specific NCD policies nationwide; in Nepal, the main activity was the implementation of the WHO Package of Essential Noncommunicable Diseases programme (WHO-PEN) [36]. Also, Nepal included in the Nepal Health Sector Programme-II an ‘Essential Healthcare Services’ programme that aimed to provide a basic package of essential medicines, free to patients, at PHC facilities and hospitals. In Mozambique, the NTD plan included a wide range of activities (e.g. mass drug administration, control activities, sanitation and health education). Still, only mass drug administration for schistosomiasis, which foreign partners funded, was implemented. Specific policies for leprosy were formulated and implemented in Nepal, also with the support of foreign aid. In contrast, Peru had no specific action plans for neurocysticercosis.

Macro-level participants in these countries identified the challenge for both NCDs and NTDs, namely, their low capacity for decision-making and planning, as well as unrealistic objectives and priorities for the PHC level and a lack of an intersectoral vision beyond the health sector.

[…] policies and guidelines are one thing, and the other is the practical dimension […]. I feel that there is a need to have a greater involvement and commitment at all levels starting from the top-level decision-makers. These are very important diseases [referring to NCDs], but the attention they are having is still inadequate. I know that the country has no money, but these [referring to NCDs] should have more priority […], without funds we are unable to have satisfactory results. (Ministry of Health, Mozambique)

The problem is not the doctor who treats and refers the patient, the problem is that when the patient returns to the community, he or she becomes sick again because the risk factors continue. This part is not only from the Ministry of Health, but also from the other sectors. The action must be done by calling other sectors. Regions would have to determine the actions because they are closer to the problem and the affected population. (Opinion Leader, Peru)

### Financing

In Mozambique, attention to both NCDs and NTDs depended on donor funding. An opinion leader highlighted the fact that the available funding was insufficient even to cover ‘basic activities’ of the departments unless a donor decided to support them financially. In Nepal, taxation on tobacco and alcohol products constituted the main funding source for NCD services; however, no specific lines in the public budget existed for attending to diabetes or hypertension. The government and donors funded Nepal’s Leprosy Control Programme. Peru had specific budget lines for ‘treatment and control’ of diabetes or hypertension, part of a ‘budgeting for results’ programme funded with general revenues. However, because it was not part of the NTD strategic plan, funding to address neurocysticercosis was not in the budget allocation for NTDs in 2017. In Peru, a participant mentioned that the PHC level was still significantly underfunded. Overall, financing for policy and programmatic responses to the selected diseases was insufficient in all countries. Moreover, donors in Mozambique still play an essential role in funding these activities.

In the public system, people in Mozambique usually paid about US$0.10 for an appointment and medicines. However, most patients with diabetes or hypertension self-reported having financial problems due to their diseases. Some had to buy their medicines in private pharmacies when medication was insufficient or unavailable in public facilities. The Leprosy Control Programme in Nepal fully covered free leprosy treatment, so none of those interviewees reported financial hurdles due to leprosy. In Nepal, the publicly funded Essential Health Care Services programme covers the cost of managing people with diabetes and hypertension, providing limited types of medicines, free at public facilities. However, some patients experienced financial problems when medicines were not available, or they incurred high transportation costs to get to hospitals. The public sector in Peru covers costs through the public health insurance programme, *Seguro Integral de Salud* (SIS). Nevertheless, half of the patients with hypertension and most patients with diabetes or neurocysticercosis related financial problems to their diseases.

We don’t have money for basic activities of the department unless a partner with a given interest decides to provide some amount […]. (Ministry of Health, Mozambique)

I spent lots of money to manage the disease, and even I sold my land. (Person with NCD, Nepal)

Sometimes I don’t have money to buy medicines and I have to stop buying my groceries to be able to buy medicines or, in any case, I buy the medicines when there is money. (Person with NCD, Peru)

### Data and information system

None of the countries had a nationwide surveillance system for all NCDs. Peru started a surveillance system for diabetes in 2011, present and still being scaled up, but with interruptions. For the selected NTDs in Mozambique and Peru, notification is not mandatory. According to the interviewees, a national system for leprosy surveillance in Nepal is functioning, with a Web-based reporting system and active surveillance in endemic areas. Although the implementation of Mozambique’s Health Management Information System (HMIS) started in 2016, PHC providers were still recording patient information manually in paper files. In Nepal, information on diabetes and hypertension was recorded in the same registry used for other diseases, while for leprosy, the registry of patients’ information operated well using the HMIS. In Peru, health-care workers used the Health Information System and the FUA-SIS form (a health services form called ‘Formato único de atención’ in Spanish, only for patients affiliated to SIS) to record patient diagnoses. Still, this system contains very general patient information, including weight, height and blood pressure measurements.

PHC facilities in all countries reported that the poor management of information created challenges regarding planning and requesting resources to respond to the population’s needs. According to a key opinion leader in Peru, improving the availability of high-quality data on the disease burden would help make better projections possible and justify a higher budget, to meet the increasing number of patients and their needs.

[…] schistosomiasis has very low [quality] data, I do not know if the problem is that people can not diagnose schistosomiasis or are not able to record it. (Health-care worker, Mozambique)

I had a notebook that suddenly disappeared. Everything changed when the management [of the PHC facility] also changed, but I’ve already made my new notebook, I’m going to start filling it in again […]. I have a notebook for diabetes and hypertension, but not for neurocysticercosis. Everything is in notebooks, not in a digital system. (Health-care worker, Peru)

### Health-care workers

Mozambique had no guidelines for diabetes nor schistosomiasis, only specific guidelines for cardiovascular diseases, developed in 2011 at the PHC level. Consequently, health-care professionals self-reported that they based their decisions on their own experience. In Nepal, the WHO-PEN protocol for the management of diabetes and hypertension in PHC was available in the facilities that had implemented the WHO-PEN programme but not yet available in the studied facilities. For leprosy, clinical guidelines were available at PHC facilities. In addition, clinical guidelines for the detection and management of diabetes, hypertension and neurocysticercosis were available in Peru. Still, these were mainly based on international guidelines and not adapted to the local context nor widely disseminated at the PHC level.

All countries lacked adequate training in diabetes and hypertension. Most medical and nonmedical health-care workers mentioned that their main source of training was their undergraduate studies. Health-care workers in Mozambique said that the only training for prevention and management of diabetes and hypertension they had received was during their professional training. Consequently, not all felt confident managing these conditions, especially regarding preventive measures and diet recommendations for patients with diabetes. However, they felt more confident managing schistosomiasis cases, even though some providers stated that the training they received was insufficient. Though PHC workers in Nepal had inadequate training on diabetes and hypertension, some training on the management of NCDs has started in areas where the WHO-PEN package is implemented. Nonetheless, according to some health-care workers, training for leprosy management last occurred a decade ago. In Peru, doctors and some nurses are the only ones who receive training at the PHC level from the regional health office, but not technicians, the main workforce in PHC. Some health-care workers said that the last training they received occurred ten years ago, with others stating that must travel to a large city and finance their training on NCDs by themselves. Moreover, they had not received appropriate training for preventing, diagnosing or managing neurocysticercosis despite its being an endemic Peruvian disease. Due to these training limitations, health-care workers in Peru felt more confident managing hypertension than diabetes and neurocysticercosis.

[…] we don’t have anything written to guide our daily practice, what we know is what we learned during our pre-service training and is based on our experience […]. (Health-care worker, Mozambique)

[…] trainings for health-care workers on management of diabetes and hypertension are inadequate and regular trainings for case detection, management, and proper counselling are necessary […], only health-care workers in upper-level centres of accessible areas like Kathmandu and Lalgadh receive training, and adequate training is lacking for health-care workers at PHC level. (Local health office, Nepal)

I haven’t had a training for ten years […]. Personally, I read books on diabetes and hypertension to at least have some knowledge. (Health-care worker, Peru)

### Medical technologies

In Mozambique and Peru, all PHC facilities had a glucometer but not all the required consumables to measure blood glucose levels. In Nepal, glucometers were not available, but PHC facilities had laboratory equipment for glucose testing. Such laboratory equipment was present in PHC facilities in Mozambique and Peru, but none had the necessary reagents. All the facilities in the three countries had at least one functioning device to measure blood pressure. In Mozambique, the number of automated sphygmomanometers available was insufficient, and all countries had problems regarding the availability of batteries or calibrated devices.

All PHC facilities in Mozambique had the diagnostic tests and necessary consumables for schistosomiasis. Only one facility in Peru had the equipment and consumables to look for the parasite’s eggs in stools. However, for both leprosy and neurocysticercosis, the additional challenge was that diagnostic tests require referral to higher levels of the health system. For neurocysticercosis, western blot tests and computerised tomography scans are only possible at hospitals. Although clinical symptoms are the main basis for a diagnosis of leprosy, a skin smear microscopy test sometimes needed for confirmation is not performed in PHC.

Availability of medicines remains a challenge in all countries (Table 3). Metformin was found in all PHC facilities in Mozambique and Nepal and half of the facilities in Peru. Glibenclamide was only available in Mozambique and half of the facilities in Peru but not in Nepal. One of four PHC facilities in Peru and a private pharmacy had insulin. PHC facilities and private pharmacies in Mozambique and Nepal had no insulin. Regarding hypertension, all PHC facilities in Mozambique and Nepal had amlodipine, and most public pharmacies in Peru had either amlodipine or nifedipine. Additionally, half of PHC facilities in Mozambique and all public pharmacies in Nepal had at least one medicine from the beta-blocker group, available in only one PHC facility in Peru. Angiotensin-converting enzyme inhibitor was available in all PHC facilities in Mozambique and Peru but not in Nepal, where only private pharmacies had enalapril for sale. Thiazide diuretics (hydrochlorothiazide) were found in most PHC facilities in Mozambique and Peru, but only in private pharmacies in Nepal.

**Table 3.** Availability of medicines in the selected PHC facilities

	Mozambique					Nepal				Peru			
Medicines by disease	Public (n = 4)			Private (n = 5)		Public (n = 2)		Private (n = 4)		Public (n = 4)		Private (n = 2)	
	n		%	n	%	N	%	N	%	n	%	n	%
**Diabetes**													
Human insulin (DNA recombinant origin)	0		0%	0	0%	0	0%	0	0%	1	25%	1	50%
Isophane insulin (NPH)	0		0%	0	0%	0	0%	0	0%	1	25%	0	0%
Metformin	4		100%	5	100%	2	100%	0	0%	2	50%	2	100%
Glibenclamide	4		100%	5	100%	0	0%	0	0%	2	50%	1	50%
**Hypertension**													
***Calcium channel blockers***													
Amlodipine	4		100%	0	0%	2	100%	4	100%	2	50%	1	50%
Nifedipine	4		100%	0	0%	0	0%	4	100%	3	75%	0	0%
***Beta-blockers***													
Atenolol	2		50%	5	100%	2	100%	4	100%	0	0%	0	0%
Carvelidol	0		0%	0	0%	0	0%	0	0%	1	25%	0	0%
***Angiotensin-converting enzyme inhibitors***													
Captopril	0		0%	2	40%	0	0%	0	0%	4	100%	2	100%
Enalapril	4		100%	4	80%	0	0%	4	100%	3	75%	2	100%
***Thiazide diuretic***													
Hydrochlorothiazide	4		100%	3	60%	0	0%	4	100%	3	75%	0	0%
**Schistosomiasis**													
Praziquantel	4		100%	3	60%	-	-	-	-	-	-	-	-
**Leprosy**													
Clofazimine	-		-	-	-	2	100%	0	0%	-	-	-	-
Dapsone	-		-	-	-	2	100%	0	0%	-	-	-	-
Rifampicin	-		-	-	-	2	100%	0	0%	-	-	-	-
Prednisolone	-		-	-	-	2	100%	4	100%	-	-	-	-
**Neurocysticercosis**													
***Antiparasitic drugs***	-		-	-	-	-	-	-	-				
Albendazole	-		-	-	-	-	-	-	-	4	100%	2	100%
Mebendazole	-		-	-	-	-	-	-	-	4	100%	2	100%
Niclosamide	-		-	-	-	-	-	-	-	0	0%	0	0%
Praziquantel	-		-	-	-	-	-	-	-	0	0%	0	0%
Pyrantel	-		-	-	-	-	-	-	-	0	0%	1	50%
***Corticosteroids***													
Dexamethasone	-		-	-	-	-	-	-	-	4	100%	2	100%
Prednisone	-		-	-	-	-	-	-	-	2	50%	2	100%
***Anticonvulsants***													
Carbamazepine	-		-	-	-	-	-	-	-	3	75%	2	100%
Phenobarbital	-		-	-	-	-	-	-	-	1	25%	0	0%
Phenytoin sodium	-		-	-	-	-	-	-	-	1	25%	0	0%
Valproate	-		-	-	-	-	-	-	-	0	0%	0	0%
Ethosuximide	-		-	-	-	-	-	-	-	0	0%	0	0%
Gabapentina	-		-	-	-	-	-	-	-	0	0%	2	100%
Lamotrigine	-		-	-	-	-	-	-	-	0	0%	0	0%
***Other psychotropic/ benzodiazepines***													
Clonazepam		-	-	-	-	-	-	-	-	0	0%	1	50%
Diazepam		-	-	-	-	-	-	-	-	4	100%	0	0%
Lorazepam		-	-	-	-	-	-	-	-	0	0%	0	0%

*Analysis includes all doses and forms combined.

** The hyphen means that the fieldworkers did not ask about this medicine because it was not for one of the target diseases in that country.

Treatment for schistosomiasis in Mozambique is available in the public health system, where all PHC facilities provided this treatment. In Nepal, medicines through the leprosy programme are also highly available in the public sector. However, except for carbamazepine to control seizures, medicines for neurocysticercosis, antiparasitic drugs and corticosteroids are only partially available in PHC facilities in Peru because the treatment is provided only at hospitals.

From the collected data, we know that the availability of some NCDs medicines at public pharmacies was ‘irregular’ and ‘hard to predict’ throughout the year. Many patients who did not find their medicines in the health facility’s pharmacy had to either buy them in the private sector or stopped taking their treatment until the PHC pharmacy had stock again. Sometimes, they could find their medication only in pharmacies in large cities.

**Figure 1.**
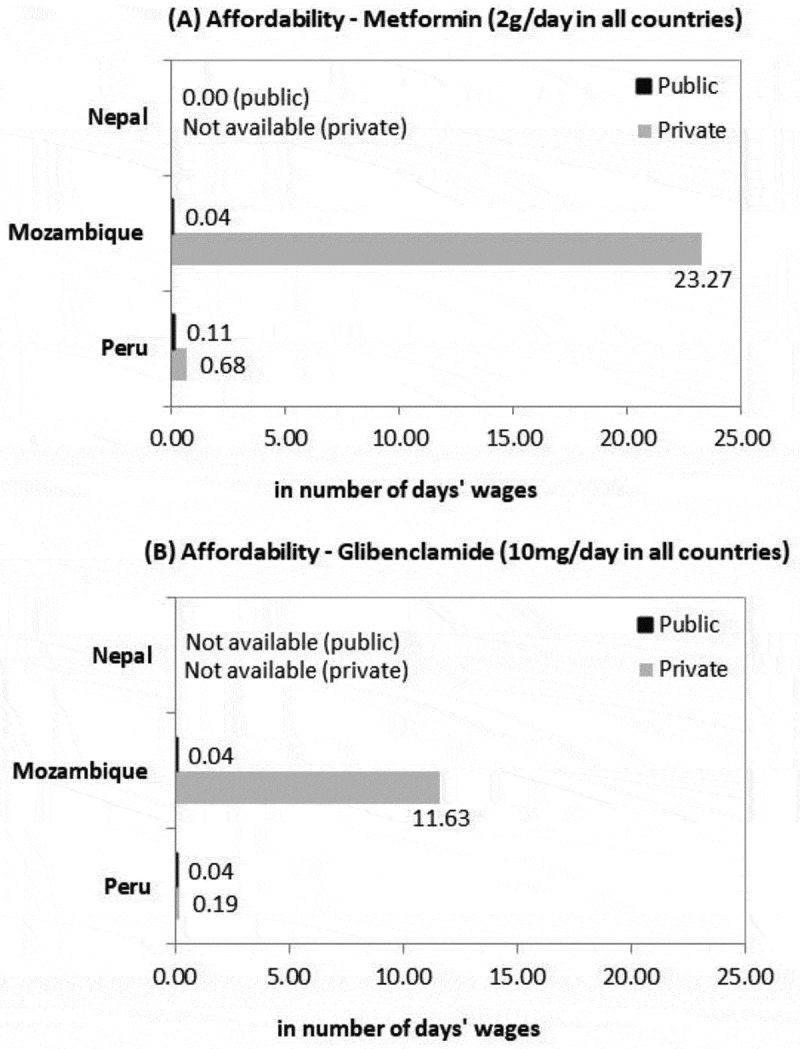
Affordability of medicines for diabetes in the selected sites

**Figure 2.**
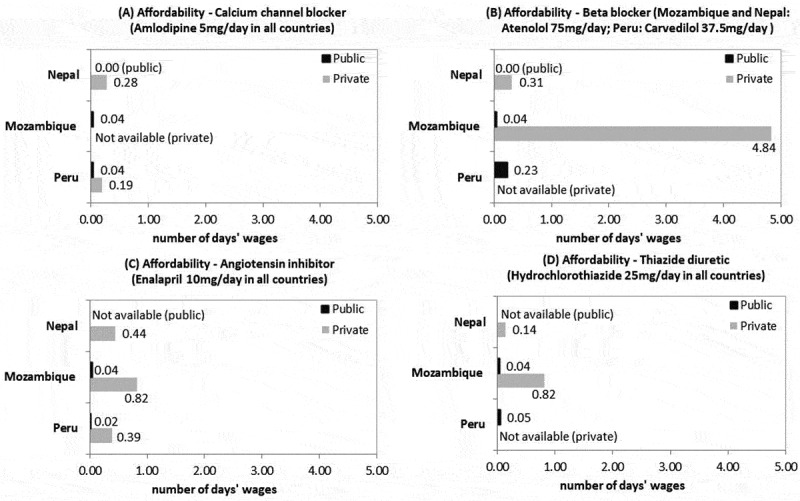
Affordability of medicines for hypertension in the selected sites

The other challenge regarding medicines is affordability. In the public health system, except for insulin, purchasing medication requires less than one day’s wages, meaning that these medicines are affordable if they are available (Figs.1**–**2). Medicines for NCDs were affordable in the private sector, except for metformin (23.3 days’ wages), glibenclamide (11.6 days’ wages) and atenolol (4.8 days’ wages) in Mozambique. Peru was the only country with available isophane insulin at one PHC facility and one private pharmacy. The treatment with isophane insulin required between 12.6 days’ wages (public pharmacy) and 19.3 days’ wages (private pharmacy), while human insulin, available only at a public facility, needed almost 31 days’ wages.

**Figure 3.**
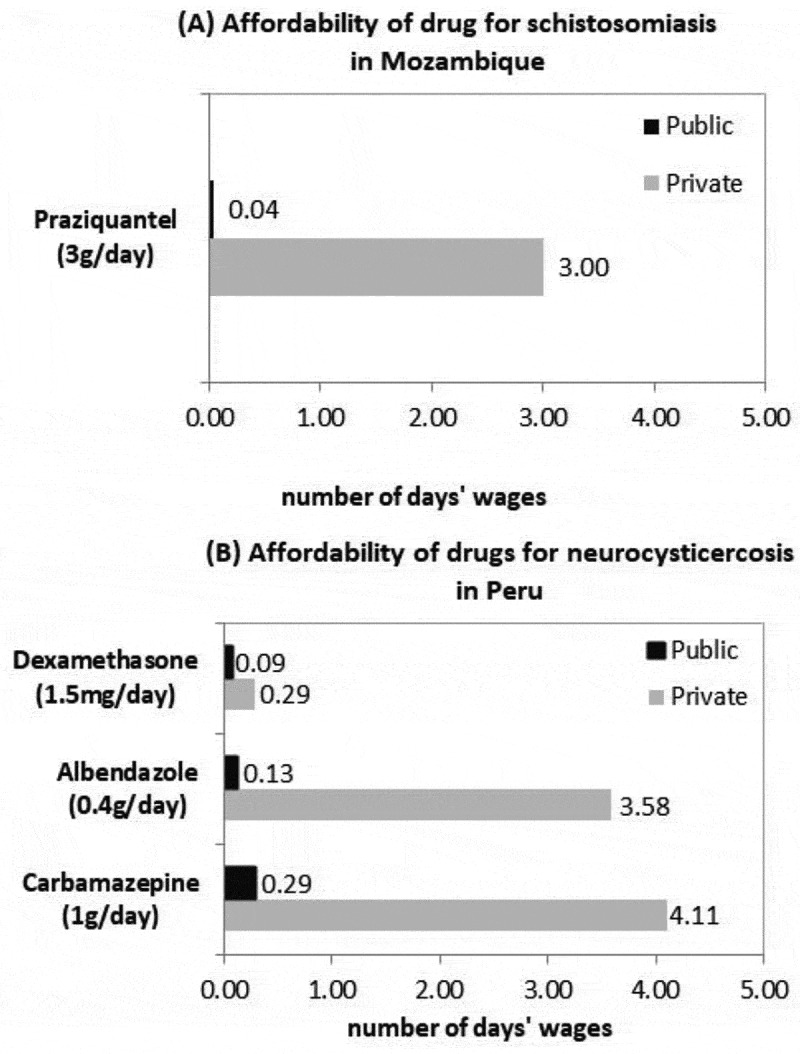
Affordability of drugs for schistosomiasis (Mozambique) and neurocysticercosis (Peru), in number of days’ wages

Similarly, the treatment for NTDs is unaffordable when patients must buy their medicines at private pharmacies (Figure 3). For schistosomiasis in Mozambique, praziquantel is affordable in the public sector but can be as high as 3 days’ wages in a private pharmacy. For neurocysticercosis, in the private sector in Peru, albendazole (3.6 days’ wages) and carbamazepine (4.1 days’ wages) were unaffordable, though they were affordable at public pharmacies. Meanwhile, the treatment for leprosy in Nepal is entirely free and available at PHC facilities.

The main constraint, I would not say main, but that turns out to be difficult that does not depend much on me, is the patient’s lack of medication. […] I ask, why are you not taking [medicines], why? ‘Haaa, I have no money to buy’, so I’m pretty much helpless. I cannot send them to the pharmacy because it does not have medicines, the patient has no medicine, no money to buy certain medicines. […] we have hardly had all of them [medicines for NCDs]. (Health-care worker, Mozambique)

In terms of access to medicines, due to logistical problems, they may not have access to medicines for periods of time, […] access is not guaranteed 100%, there are only 6 to 7 months of the year, but then they run out. […] the transport of medicines is another constraint, especially in remote areas. […] the laboratories do not have biosecurity, they are tiny, they do not have washing facilities, sometimes they do not have water. (Regional health office, Peru)

### Service delivery

Many of the weaknesses described above impact the quality of health-care delivery. This study explored two areas of service delivery, access to information and referrals. These face issues of quality and how the information is provided, given the low education levels of the population in the study settings. The NCD information provided through medical consultations and leaflets mainly focused on lifestyle advice and less on treatment: e.g. increasing fruit and vegetable consumption and physical activity, smoking cessation, and decreasing sugar, salt and fried food intake. Health-care workers in all countries saw NCD-related material and information as inadequate, as was information in Peru for neurocysticercosis. Health-care professionals and users found the information for both schistosomiasis and leprosy adequate. Moreover, all patients with leprosy were satisfied with the information provided for prevention, symptomatology and treatment of their diseases, usually provided through educational classes, brochures and flipcharts, and they found it useful.

In Peru, almost all the health facilities had a flowchart showing the referral process for any disease. Most PHC workers said that the referral process works effectively, especially for guiding patients to appointments or exams at an upper level. In these cases, referral appointments are given for the next 15–30 days. In contrast, in Mozambique and Nepal, referral procedures are unclear. Health-care providers in Mozambique highlighted people sent back, apparently for not meeting the referral criteria and communication problems. Still, health-care providers at PHC could not manage them. In Nepal, all the facilities interviewed had a referral guideline for patients with leprosy, a flow chart with the referral criteria for patients with symptoms and complications of leprosy and a map showing the location of leprosy referral centres. Participants from Peru and Mozambique also highlighted challenges around available transportation to regional hospitals, especially in emergencies. Overall, these issues highlight the lack of PHC role definition in delivering services for chronic conditions.

The process of referral of analyses sometimes has some difficulties because sometimes the analyses do not arrive, or was poorly collected […], so if the patient appears to have diabetes problems today and we suspect, we can not medicate before we have a laboratory confirmation, so this is another process that we have to wait for the results to arrive and only then we can start to act. (Health-care worker, Mozambique)

Patients with diabetes or hypertension are referred by health-care worker according to their knowledge, if they feel the case is complicated, with referral note written in OPD [out patient services] card […]. In Baniyani, the main difficulty for patients when referred are transport problems, distance and inappropriate road, access and poor economic condition of patients […]. Patients are basically advised to go to Bhadrapur hospital and BPKIHS [B.P. Koirala Institute of Health Sciences], and there is no follow up of such cases. (Health-care worker, Nepal)

The most frequent problems in the referral is not having a functioning ambulance or enough fuel, sometimes it operates without oxygen, cardio-resuscitator, or oximeter […]. (Health-care worker, Peru)

## Discussion

This study highlights the complexity and challenges of addressing chronic care needs for NCDs and NTDs at the PHC level across three LMICs. Using different chronic diseases as ‘tracer conditions’ and a multilevel health system assessment methodology, we found that PHC preparedness in Mozambique, Nepal and Peru is still inadequate and insufficient in the context of various WHO building blocks. Our study especially highlights that health systems in LMICs fail to go beyond specific vertical programmes or health conditions in moving from the provision of acute to chronic care and from single to multiple conditions [37,38].

Our results indicate that the ambitious policies in place in these countries are not matched by technical, administrative and financial resources, except for those related to leprosy in Nepal, where there is a vertical donor-funded programme. However, vertical approaches in the study countries have not been sufficient to strengthen the health systems. Moreover, policies do not address intersectoral issues, and adequate and sustainable financing is a challenge. Although essential medicines are affordable or free in the public sector, they are not widely available at PHC facilities. Therefore, individuals end up having high out-of-pocket expenditures in private pharmacies. This is the case for medicines for NCDs in Mozambique or NTDs in Peru; in those countries, some medicines are unaffordable, as the monthly cost exceeds the one-day’s-wage threshold [39]. Although insulin is only in Peru and not available in other countries, it is costly at up to 31 days’ wages.

The availability of functioning equipment and consumables is a significant problem in the three countries. When diagnostic tests are unavailable, transportation costs to another laboratory also increase patient’s out-of-pocket expenses. Essential equipment, such as blood pressure measurement devices and glucometers, with their respective consumables, were not available or functioning in most facilities. Also, clear procedures for referrals are lacking, and several problems impede transportation to a higher level for emergencies. Compounding these are clinical guidelines and training that are non-existent, insufficient or not adapted to local contexts and PHC. Adding to the challenges, are inadequate and insufficient education and information for patients, especially regarding pharmacological treatment. Finally, patients’ data and information systems do not meet the needs of the individuals or the health systems; they are still limited, and registries are mainly paper-based files that do not allow for individualised management or planning for necessary resources.

Although Mozambique, Nepal and Peru are very different LMICs in terms of cultural, socio-economic and health-system characteristics, they face similarities and critical challenges to overcome at the PHC level in areas with vulnerable populations. Chronic conditions negatively affect people’s lives, and the economic consequences of health implications for productivity losses and higher out-of-pocket expenses further challenge effective high-quality care [40]. Improving PHC is essential for universal health coverage in LMICs [41,42] and achieving SDG 3 [12]. This study shows that PHC is currently insufficiently prepared to manage NCDs and NTDs in these countries.

Previous studies highlight the challenges of managing chronic diseases in LMICs [22,43–46]. We applied a mixed-methods approach, previously implemented to assess the response of health systems to chronic diseases, using a systematic methodology adaptable to LMICs [20,23]. In contrast to other methods, e.g. the Service Availability and Readiness Assessment [47], we used a methodology for studying the health-system barriers through various sources of information and the perspectives of policy makers, health authorities, opinion leaders, health-care providers and patients. Therefore, the strength of our study is the depth of information, combining different views from participants at three health-system levels in three very different LMICs, using a set of ‘tracer conditions’. Two of these conditions, diabetes and hypertension, have also been proposed as ‘tracer conditions’ for use with the Primary Health Care Impact, Performance and Capacity Tool (PHC-IMPACT) for monitoring PHC capacity in high- and middle-income countries [19]. Unlike the PHC-IMPACT, our study includes an NTD with chronic sequelae endemic to each setting. The tool used allowed us a comprehensive view of the problem through various elements of the health system. Moreover, future studies could include more topics to improve the breadth of topics covered, such as the availability of human resources, responsiveness and equity.

However, the approach we followed presents different challenges. First was the identification of informants at different levels of the health system, who could provide the necessary information. Another challenge was representativeness; such an approach favours depth over breadth, requiring information collection from different sources and informants to triangulate results. Also, by triangulating information at different levels, we corroborated the chosen facilities as unexceptional and reflecting many of the problems found at PHC in the selected settings. Finally, the fieldwork team needed specific training in qualitative methods of data collection, which are unlike methods that use a standard structured questionnaire.

The overarching finding of this study is that current systems fail to provide adequate chronic care for the populations they serve. A major need is to leapfrog advancements at the PHC level in LMICs to address the so-called ‘quality gap’ [48–50]. In addition, the 40^th^ anniversary of the Alma-Ata Declaration highlighted the need to focus on social aspects of health and disease [8,51] and build links and trust [52] between populations and PHC, to ensure that the health system is responsive to their needs. There is also the need to have stronger integration between PHC and the community [53] and their environment. PHC services in many LMICs face multiple challenges, and they must adapt to accommodate the growing demands for chronic care, guaranteeing high-quality care and continuity of care for the populations they serve, in the face of changing disease patterns and population needs.

## Conclusion

By using different NCDs and NTDs as ‘tracer conditions’, this study shows that PHC preparedness in Mozambique, Nepal and Peru is still insufficient to adequately address chronic diseases, with weaknesses in all six WHO building blocks. Beyond tangible elements that the health system and PHC must deliver, health systems also need to address the issue of guaranteeing high-quality care to the populations they serve. Strengthening primary care is urgently needed, to guarantee the delivery and continuity of high-quality care for patients with chronic conditions, and more so in LMICs, as they move towards the goal of achieving universal health coverage.

## Supplementary Material

Supplemental Material

## Paper context

Forty years after Alma-Ata, low- and middle-income countries still need to strengthen primary health care (PHC) and improve preparedness for chronic diseases. Mozambique, Nepal and Peru share similar challenges in the preparedness of care provision for multiple diseases. Besides the need for intersectoral strategies and guaranteeing sufficient funding for chronic diseases at the PHC level, it is crucial to ensure availability and accessibility to essential medicines, equipment and consumables, clinical guidelines, and appropriate information systems.
