# Supplementary material for: Forty years after Alma-Ata: primary health-care preparedness for chronic diseases in Mozambique, Nepal and Peru
Source: Glob Health Action. 2021 Sep 27;14(1):1975920. doi: 10.1080/16549716.2021.1975920 (PMC8477950; doi:10.1080/16549716.2021.1975920)
Supplement: Supplemental Material [file ZGHA_A_1975920_SM6818.docx]

## Supplementary Material 1. Documents and secondary information collected per country

| **Mozambique** |
| --- |
| National Strategic Plan for Prevention and Control of NCD 2008-2014. |
| Five Year Government Plan 2010-2014 [Plano Quinquenal do Governo 2010-2014]. |
| Guidelines for the diagnosis, treatment and follow up of hypertension and other cardiovascular health factors for the primary health care, 2011. |
| National Integrated Plan for the Control of Neglected Tropical Diseases, 2013-2017 [Plano Nacional Integrado de Controlo de Doenças Tropicais Negligenciadas, 2013-2017]. |
| National Strategic Plan of the Health Sector, 2014-2019. [Ministério da Saúde. Plano Estratégico do Sector da Saúde PESS 2014-2019]. |
| Five Year Government Plan 2015-2019 [Plano Quinquenal do Governo 2015-2019]. |
| Department of Other Infectious Diseases Annual Activities Report, 2017. |
| **Nepal** |
| Nepal Health Sector Programme-II (NHSP-II), 2010 – 2015. |
| National Strategy to Sustain Quality Leprosy Services and Further Reduce the Disease Burden due to Leprosy in Nepal, 2011-2015. |
| National Health Insurance Policy-2013 (NHIP), 2013. |
| Leprosy control programme. National implementation guideline, 2013. |
| Guideline for Leprosy Programme, 2013. |
| National Health Policy, 2014. |
| Multisectoral Action Plan for the Prevention and Control of Noncommunicable Diseases (2014-2020). |
| Constitution of Nepal, 2015. |
| Leprosy Annual Report 2015/16. |
| Nepal Health Sector Strategy (NHSS), 2015-2020. |
| Package of Essential Noncommunicable Disease programme. WHO-PEN Implementation Plan 2016, Nepal health facility survey 2015. Final report. |
| Guidelines for Leprosy Elimination Campaign in Sub National Level 2073, 2016. |
| 14th periodic plan (FY 2073/74-2075/76)(2016/17-2018-19) |
| Leprosy control program guidelines, 2073/2074, 2016/2017. |
| Guideline for leprosy elimination at district level, 2016/17. |
| National health policy (draft), 2017. |
| **Peru** |
| Model of Integral Health Care Based on the Family and Community [Modelo de Atención Integral de Salud Basado en Familia y Comunidad], 2011 |
| National Plan to Strengthen Primary Health Care 2011-2021 [Plan Nacional de Fortalecimiento del Primer Nivel de Atención 2011-2021]. |
| National Policy on Environmental Health 2011-2020 [Política Nacional de Salud Ambiental 2011-2020]. |
| Institutional Strategic Plan 2012-2016 of the Ministry of Health [Plan Estratégico Institucional 2012-2016 del Ministerio de Salud] - R.M. No. 666-2013/MINSA. |
| Sanitary Directive for the Epidemiologic Surveillance of Diabetes in Health Facilities [Directiva Sanitaria para la Vigilancia Epidemiológica de Diabetes en Establecimientos de Salud], 2014 |
| Sanitary Directive for epidemiological surveillance of zoonotic diseases, accidents caused by poisonous animals and epizootic diseases [Directiva Sanitaria para la Vigilancia Epidemiologica de Enfermedades Zoonoticas, Accidentes por Animales Pozoñosos y Epizootias], 2015. |
| Technical Guideline: Practical Clinical Guideline - Neurocysticercosis [Guía Técnica: Guía de Práctica Clínica – Neurocisticercosis], 2015 |
| Technical Guideline: Practical Clinical Guideline for the Diagnosis, Treatment and Control of Hypertension [Guía Técnica: Guía de Práctica Clínica para el Diagnóstico, Tratamiento y Control de la Enfermedad Hipertensiva], 2015. |
| Technical Document: Nutritional Consultation for the Prevention and Control of Diabetes Mellitus Type 2 of Young Adult, Adults and Elderly [Documento Técnico: Consulta Nutricional para la Prevención y Control de la Diabetes Mellitus Tipo 2 de la Persona Joven, Adulta y Adulta Mayor], 2015. |
| Technical Guideline: Practical Clinical Guideline for the Diagnosis, Treatment and Control of Diabetes Mellitus Type 2 in Primary Health Care [Guía Técnica: Guía de Práctica Clínica para el Diagnóstico, Tratamiento y Control de la Diabetes Mellitus Tipo 2 en el Primer Nivel de Atención], 2016. |
| Political and Strategic Guidelines for the Prevention and Control of NCDs for the period of 2016-2020 [Lineamientos de Políticas y Estrategias para la Prevención y Control de Enfermedades No Transmisibles 2016-2020]. |
| Multiannual Sectorial Strategic Plan 2016-2021 [Plan Estratégico Sectorial Multianual 2016-2021]. |
| Technical Guideline: Clinical Practice for the Diagnosis, Treatment and Control of Diabetic Foot [Guía Técnica: Guía de Práctica Clínica para el Diagnóstico, Tratamiento y Control del Pie Diabético], 2017. |
| Institutional Strategic Plan 2017-2019 of the Ministry of Health [Plan Estratégico Institucional 2017-2019 del Ministerio de Salud] - RM No. 950-2016 / MINSA. |
| Epidemiological bulletin of Peru, Vol.27-SE36, 2018 [Boletín Epidemiológico del Perú, Vol.27-SE36, 2018]. |

# Supplementary Material 2. Data collection and analysis matrix

| **WHO building block and topic of study** | **Core Information for each building block and topic** | **Source and type of informant** | **Main questions on each topic of study** |
| --- | --- | --- | --- |
| **1.Building Block: Governance** | | | |
| 1) National health plans, policies, strategies or action plans for preventing and managing NCDs and NTDs | 1a. Inclusion of (DISEASE) in the national health plan (policies, strategies or action plans) and/or your national development agenda. | - Literature - Policy documents | **Review** of documents and secondary information to identify if NCDs and NTDs are included in the national health plans (policies, strategies or action plans) and/or national development |
|  | 1b. Inclusion of (DISEASE) in the national health plan, existence of a policy/strategy/action plan for prevention and management of (DISEASE) and its implementation | - Literature - Policy documents - Interviews at the macro-level and meso-level:   - Ministry of Health (MH)   - Opinion leaders (OL)   - Regional/Local health office (RHO)   - Head of facilities (HF) | **Review** of documents and secondary information to identify policies, programmes and activities related to the selected NCDs and NTDs that have been developed at the national, regional and community level of the selected sites.  **Question (MH, OL, RHO, HF):** In your opinion, which are the main policies and programs that exist at a national, regional or local level for delivery of care of (DISEASES)? List them.  **Question (MH, OL, RHO, HF):** In your opinion, how has the implementation of the policy, strategy or action plan for prevention and management of (DISEASE) been? (Linked with findings from literature and policy documents) |
| 2) Organizational structure within the Ministry of Health related to the selected NCDs and NTDs | 1c. Existence of a functioning unit/branch/department with resources in the Ministry of Health or equivalent with responsibility for (DISEASE) | - Literature - Policy documents - Interviews at the macro-level and meso-level:   - Ministry of Health (MH)   - Regional/Local health office (RHO) | **Review** of documents and secondary information to identify units and/or actors in charge of NCDs and NTDs  **Question (MH, RHO):** Within this institution, is there a functioning unit/branch/department with resources responsible for (DISEASE)? If yes, how well does this group work? |
| **2. Building Block: Financing** | | | |
| 3) National budget line for the selected NCDs and NTDs | 2a. Specific budget line within the national health budget devoted to the prevention and management of (DISEASE) | - Literature - Interviews at the macro-level and meso-level:   - Ministry of Health (MH)   - Regional/Local health office (RHO) | **Review** of documents and secondary information to identify information on available budget for the prevention and management of (DISEASES).  **Question (MH, RHO):** Is there a specific budget line within the national health budget devoted to the prevention and management of (DISEASE)? If Yes, please detail. |
| 4) Funding of the selected NCDs and NTDs and financial consequences for patients | 2b. The cost of managing people with (DISEASE), its coverage through a publicly funded system or public insurance and financial consequences for patients | - Literature - Interviews at the macro-level, meso-level and micro-level:   - Ministry of Health (MH)   - Regional/Local health office (RHO)   - Patients or caregivers (PC) | **Review** of documents to identify information on public insurance in the country and funding of the selected diseases.  **Question (MH, RHO):** Is the cost of managing people with (DISEASE) covered through a publicly funded system or public insurance? Please, comment.  **Question (PC):** At any point, has your illness created financial problems? Please, explain.  **Question (PC):** At any point, have you had to stop following the instructions of the doctor for treatment of your illness due to economic difficulties? Please, tell me, what part of the treatment you had to stop completely or partially. |
| **3. Building Block: Data and information system** | | | |
| 5) National surveillance system and registry of the selected diseases | 3a. Existence of national system surveillance and a system implemented at the PHC level for recording the cases of (DISEASE). | - Literature - Interviews at the macro-level, meso-level and micro-level:   - Ministry of Health (MH)   - Regional/Local health office (RHO)   - Head of facilities (HF)   - Health-care workers (HW) | **Review** of documents and secondary information to identify available information on national systems for registry and surveillance.  **Question (MH, RHO, HF):** Is there a national system implemented for recording the cases of (DISEASES) in the public sector? How well does this recording system work?  **Question (MH, RHO, HF):** Is there a surveillance system for patients or cases of (DISEASES) in the public sector? How well does this surveillance system work?  **Question (HF, HW):** Does a special registry of patients or cases of (DISEASES) exist in this facility?  **Question (observation on-site) (HF, HW):** Availability of: Registry of patients with (DISEASE) and Patient clinical records – Present |
| **4. Building Block: Health-care workers** | | | |
| 6) National clinical practice guidelines about prevention and management of the selected NCDs and NTDs at the PHC level | 4a. Existence and use of national clinical practice guidelines devoted to the prevention and management of (DISEASE) at the primary health-care level. | - Interviews at the macro-level, meso-level and micro-level:   - Ministry of Health (MH)   - Opinion leader (OL)   - Regional/Local health office (RHO)   - Head of facilities (HF)   - Health-care workers (HW) | **Question (MH, OL, RHW, HF, HW):** In the country, are there national clinical practice guidelines devoted to prevention and management of (DISEASE) at the primary health-care level? Please, comment.  **Question (MH, OL, RHW, HF, HW):** What is your opinion on the clinical guidelines for (DISEASE)? Inquire: problems, gaps, etc.  **Question (observation on-site) (HF, HW):** Evidence-based clinical protocol for (DISEASE) – Present  **Question (HW):** What do you usually use to guide on the most appropriate management for (DISEASE)? (e.g. clinical practice guidelines) |
| 7) Knowledge and training of health-care workers on the selected diseases | 4b. The health-care workers receive appropriate knowledge and training of health-care workers about prevention, diagnoses and management of patients with (DISEASE). | - Interviews at the meso-level and micro-level:   - Regional/Local health office (RHO)   - Head of facilities (HF)   - Health-care workers (HW) | **Question (RHO, HF):** Do health professionals receive special training on the management of (DISEASE)? Please explain (what type of training, where is it provided, by whom and when, e.g. medical/nursing school, special training, etc.)  **Question (HF, RHO):** Do you believe that the health-care staff is sufficiently trained to treat patients with (DISEASE)? Explain.  **Question (HF, HW):** Are health-care workers receiving appropriate training for prevention, diagnoses and management of patients with (DISEASE)? Ask about what kind of training.  **Question (HW):** Have you received special training to address patients with (DISEASE)? If Yes, please indicate what training you have received.  **Question (HW):** Do you believe that your training is sufficient to treat patients with (DISEASE)? Explain your response. |
| **5. Building Block: Medical technologies** | | | |
| 8) Availability and price of tests and consumables for the selected diseases* | 5a. FOR NCDs: Devices and consumables/ reagents (if applicable) to measure blood glucose concentration, glycosylated hemoglobin, blood pressure, creatinine, microalbuminuria, lipid profile, among others, at the primary health-care centres.  FOR NTDs: Devices and/or consumables to diagnose and to monitor (selected NTD) at the primary health-care level.* | - Interviews at the meso-level and micro-level:   - Regional/Local health office (RHO)   - Head of facilities (HF) or health-care worker (HW)   - Laboratories (LF) | **Question (RHO):** What are the main problems regarding diagnostic tools for (DISEASE) in the PHC?  **Question (observation on-site) (HF/HW, LF):** Are the following instruments and supplies available for the management of patients with (DISEASE)? Table: Instruments and supplies provided in this facility for the care of patients with (DISEASE). (Information in the table includes: Name of instrument/supply/test, equipment available, equipment functioning, reagents available, price of the test).  **Question (HF/HW, LF):** Does the stock of reagents usually cover 100% of the monthly needs?  **Question (LF):** What difficulties do you believe patients with (DISEASE) often experience in accessing to their exams? |
| 9) Availability and price of essential medicines for the selected diseases** | 5b. Medicines of the essential drug list for (DISEASE) at the primary health-care level: FOR NCDs: insulin (different types), metformin, glibenclamide, gliclazide, calcium channel blocker, beta-blocker, angiotensin-converting enzyme inhibitor, and thiazide diuretics. FOR NTDs: (according to the disease treatment).** | - Interviews at the meso-level and micro-level:   - Regional/Local health office (RHO)   - Pharmacies (PF)   - Health-worker (HW) | **Question (RHO):** What are the problems that the supply of medicines for (DISEASE) faces?  **Question (HW):** What difficulties do you believe patients with (DISEASES) often experience in receiving treatment for their disease? Discuss availability and affordability.  **Question (PF):** Are the following medicines and supplies available to manage patients with (DISEASE)? Table: Medicines and supplies available at this pharmacy for the care of patients with (DISEASES). (Information in table includes: name of medicine, presentation, available or not, price for retail sale per unit)  **Question (PF):** Does the stock or medicines usually cover 100% of the monthly needs? |
| **6. Building Block: Service delivery** | | | |
| 10) Prevention of the selected diseases through patient education | 6a. Information received by patients with diabetes about prevention, symptomatology and treatment of their disease. | - Interviews at the meso-level and micro-level   - Head of facilities (HF)   - Health-workers (HW)   - Patients or caregivers (PC) | **Question (HF, HW):** In this facility, what type of information about their diseases do patients with (DISEASES) have access to? Explain.  **Question (PC):** Have you received information about prevention, symptomatology and treatment of your disease? If Yes, please describe (the information, how it was obtained, in which format and who gave the information). Did you find it useful? Please discuss.  **Question (PC):** Have you received information about healthy diet, physical activity and tobacco cessation in this facility? If Yes, please describe (the information, how it was obtained, in which format and who gave the information). Did you find it useful? Please discuss |
| 11) Referral process information and implementation | 6b. Available information about the referral process for patients with symptoms or complications of (DISEASE) and how well it works. | - Interviews at the meso-level and micro-level:   - Head of facilities (HF)   - Health-workers (HW)   - Patients or caregivers (PC) | **Question (HF, HW):** Is there any guide or detailed document on how to refer a patient with symptoms of complications of (DISEASES) to another facility? Please, comment about the topics covered and how useful is.  **Question (HF, HW):** Is the referral process for patients with symptoms or complications working effectively? Explain.  **Question (HF, HW):** What difficulties exist in the referral and counter-referral process?  **Question (PC):** Have you ever been referred from one health facility to another for treatment of your illness or due to a complication? If Yes, please specify where.  **Question (PC):** Do you remember if there were any problems getting a referral? Please, explain the problems. |

Diseases: Hypertension, diabetes mellitus, and selected NTDs (schistosomiasis in Mozambique, leprosy in Nepal and neurocysticercosis in Peru).

* For NCDs, the equipment was selected based on the Rapid Assessment Protocol proposed by Beran and Higuchi (2012) [20]. For NTDs, each country added the necessary equipment and consumables according to the management of the disease.

** Drugs can be selected according to national protocol, or international/national list of essential medicines. Each country added the medicines according to the management of the NTD in the country. For example, for neurocysticercosis, antiparasitic drugs and symptomatic medication such as antiepileptics and corticoids were included.
